# Sustainable Development Efficiency of Cultural Landscape Heritage in Urban Fringe Based on GIS-DEA-MI, a Case Study of Wuhan, China

**DOI:** 10.3390/ijerph192013061

**Published:** 2022-10-11

**Authors:** Han Zou, Yang Liu, Baihao Li, Wenjing Luo

**Affiliations:** 1 School of Civil Engineering, Architecture and Environment, Hubei University of Technology, Wuhan 430068, China; 2 Innovation Demonstration Base of Ecological Environment Geotechnical and Ecological Restoration of Rivers and Lakes, Wuhan 430068, China; 3 School of Architecture, Southeast University, Nanjing 210096, China; 4 Wuhan Planning and Design Institute, Wuhan 430014, China

**Keywords:** GIS-DEA-MI model, urban fringe, cultural landscape heritage, sustainable development efficiency, heritage preservation and utilization

## Abstract

Cultural landscape heritage refers to the rare and irreplaceable cultural landscapes recognized by UNESCO and the World Heritage Committee. It is recognized as a “common works of nature and human beings” of outstanding significance and universal value, and is a type of world heritage. Dueto construction, land isincreasingly limited in urban and rural areasin the process of urbanization, and cultural landscape heritage faces a huge threat, especially larger culturallandscapeheritagelocated at the edgesof cities. However, most of the existing studies have mainly focused on the material protection of heritage but have not paid enough attention to the non-material aspects of heritage sites, failing to reveal the inseparable nature of heritage and land. Therefore, this study takes sustainable development efficiency as its analysis tool, examines two pieces of cultural landscape heritage (the Panlongcheng site and the Tomb of the King of the Ming Dynasty) in the urban edge area of Wuhan, China as examples, innovates and establishes a multidimensional evaluation method based on the GIS-DEA-Ml model, and compares the dynamic changes of the spatial development efficiency and non-spatial development efficiency of the above two cultural landscape heritage cases. The results show that: both the spatial development efficiency and non-spatial development efficiency of Panlongcheng from 2010 to 2019 are significantly higher than that of the Tomb. This method makes up for the deficiency of traditional subjective qualitative analysis. It can be used to study the development efficiency of cultural landscape heritage more objectively and comprehensively, and promote the overall sustainable development of material and intangible cultural heritage. It can provide the basis for early decision-making and post-implementation evaluation for the preservation and utilization of cultural landscape heritage under the background of urban renewal.

## 1. Introduction

J. Jackson believed that landscapes are never a simple natural space or a natural environment, and that each landscape is the space-time place of human beings [1]. In the first half of the 20th century, German geographer Otto Schluetter put forward the theory of cultural landscapes. He believed that landscapes can be divided into two categories; one is the original landscape, and the other is the cultural landscape (the landscape changed by human activities). He thought that cultural landscape is the form of human phenomena on the ground, and should be studied as a landscape created by human beings and their labor that can reflect the culture and economy of human groups. In 1906, Otto Schluetter formally put forward the concept of “cultural landscape form”, emphasizing that landscape has not only its appearance, but also social, economic and spiritual power. It also points out the difference between cultural landscapes and natural landscapes, and requires the study of cultural landscapes as a phenomenon evolved from natural landscapes, and the form of landscapes as cultural products [2].

American geographer C. O. Sauer inherited and developed Otto Schluetter’s theory of cultural landscape. He believed that cultural landscape is “a region composed of significant forms of connection including nature and culture”. C. O. Sauer believed that a special human group, under the influence of culture, can create suitable surface features in areas that are long-lived and active. This feature is caused by the different ways in which human beings intervene with, apply and transform the environment, and is also a result of the transformation from natural landscape to cultural landscape [3]. In Sauer’s paper on landscape morphology in 1925, the natural landscape is the result of the effect of human culture on the natural landscape [4]. In the Recent Development of Cultural Geography, published by Sauer in 1927, cultural landscape was first clearly defined for the first time as “the form of human activities appended to the natural landscape” [5].

In 1992, the World Heritage Committee included cultural landscape heritage in the World Heritage List. The study of cultural landscape heritage has attracted extensive attention from scholars all over the world. David Jacques called the 1990s “the rise of cultural landscape” [6]. The distribution of word culture landscape heritage in various regions is extremely uneven. The world cultural landscape heritage in Europe accounts for more than half of the world total, which is three times that of Asia [7]. Walliams Logan presented a report at the 10th UNESCO University and Heritage Symposium with the theme of “The Cultural Landscapes in the 21st Century”, noting that “the conservation and utilization of cultural landscapes is much more smooth in Europe than in Asia” [8]. The 2004 ICOMOS report also pointed out the shortage of the word cultural landscape heritage sites in Asia [9]. Many of Asia’s heritage sites have been designated as natural heritage sites, but in fact many of the cultural associations have been obliterated, although the number of world cultural landscape heritage sites in Asia is insufficient [2]. However, Professor P. Fllower analyzed the number and value of cultural landscapes in Asia and confirmed the value of cultural landscapes in Asia [10]. In Asian world heritage sites or candidate lists, the most important are human sites, architectural monuments and religious heritage. However, the World Heritage Committee did not consider cultural landscapes, vernacular architecture, technological or agricultural sites as a whole. These belong to the category of cultural landscape sequences, leading to a missed opportunity of demonstrating Asian local spirit [2]. For the sustainable and healthy development of Asian cultural landscape heritage, Ken Taylor recommends making efforts in the following areas [11]: give a clear Asian definition of cultural landscapes, define the content of Asian cultural landscape types according to Asian values and a regional cultural landscape list, grasp the construction and practice of cultural quantitative concepts, connect nature and culture, take man as an integral part of nature, and establish a unique protection mode, so as to avoid the simple imitation of the Western national park protection system of pure nature protection. Professor Ken Taylor believes that Asia’s cultural landscapes can make a significant contribution to world heritage, and China should play a leading role [12]. Mitchell believes that China has a long history of civilization and unique man-land relationships, and covers a large part of its natural heritage. Therefore, it has special value to observe and study heritage protection from the perspective of cultural landscapes. The Asian cultural landscape perfectly reflects the interactive relationship between man and nature. It is not only a tangible cultural work, but also the result of the cultural effect of associative intangible values. In this respect, different from Western cultural landscapes, the Asian cultural landscape is a part of a “dynamic process of identity formation” [2]. Han Feng pointed out that in Eastern culture, nature and culture are an inseparable integral whole, and cultural landscape is the link between nature and culture heritage, revealing the inseparable nature of heritage, land and landscape. He once pointed out that China has difficulty understanding the concept of “cultural landscape” [13]. He believes that the landscape in the Eastern tradition itself has strong cultural attributes. Different from the West, landscape in Eastern culture is a landscape with special significance.

Some natural heritage scholars in the West regard nature and culture as two completely opposite things. In their view, human beings are not a part of nature and the landscape is not a product of culture. However, China pays attention to the overall characteristics of the landscape and believes that the protection of cultural landscape heritage not only involves simple material protection, but also involves the cultural and social dimensions. Therefore, non-material factors such as society, economy and land development and utilization need to be further considered in the protection and utilization of cultural landscape heritage and the study of sustainable development in China. Cultural landscape is the result of the interaction between human beings and nature. Its integrity and importance require us to achieve sustainable development in the management of cultural landscape heritage. The current management of China’s cultural landscape heritage takes sustainability as the basic principle and overall requirement, and coordinates multiple fields such as society, economy and environment. The effectiveness of management is directly related to the status quo of cultural landscape heritage and the possibility of sustainable development, and the effectiveness evaluation of management refers to the objective evaluation of the process, results, efficiency and rationality of project implementation under the background of management. By setting reasonable evaluation indexes, the planning, management system and implementation in the process of regional management are evaluated scientifically.

Scholars from different countries have different perspectives on cultural landscape heritage. For example, in research on the protection and utilization of cultural landscape heritage, Shizhenjia et al. explored the protection and management methods of Chinese classical royal garden cultural heritage based on three-dimensional digital technology, which improved the management efficiency of cultural landscape heritage and promoted the sustainable development of cultural landscape heritage [14]. Xuhui Wang et al. constructed the ecological security model of ancient capital landscape based on cellular automata theory [15]. Alessandra Capuano explored more advanced Roman archaeological sites and urban sustainability based on sustainable concepts [16]. Janjira Sukwai used GIS and computer-generated 3D modeling to support a visual integrity assessment of change management in the Chiang Mai Historical City buffer zone [17]. In the study of the sustainable coordinated development of cultural landscape heritage and tourism, UNESCO launched the World Heritage Sustainable Tourism Program in 2001 in response to the opportunities and threats posed by tourism activities in World Heritage sites [18]. Joanne Connell et al. studied the monitoring of local government planning in sustainable tourism planning in New Zealand [19]. Muhamment Yasarata et al. studied the coexistence of sustainable tourism development and politics [20]. The contradiction between tourism development and sustainable heritage has always been a topic of academic debate. Di Zuo et al. explored the relationship between tourism, residents’ practice and traditional cultural landscapes in cultural heritage sites by taking Hongcun Village in China as an example, and proposed the possibility of capital-driven and sustainable development and local cultural balance, which is of great significance to the sustainable development of cultural heritage [21].

The above research emphasizes the sustainable development of cultural landscape heritage, but mostly only focuses on the protection of cultural landscape heritage, cultural landscape heritage and the sustainable development of tourism. The sustainable development of cultural landscape heritage and the surrounding environment research perspective is insufficient, given the lack of quantitative research on the efficiency of the sustainable development of cultural landscape heritage. In addition, cultural landscape heritage is mostly located in urban edge areas, and the protection, utilization and sustainable development of the cultural landscape heritage are threatened by urban expansion. Therefore, it is particularly important to study the coordination and sustainable development of cultural landscape heritage sites and the surrounding environment, and cultural landscapes should be included in current territorial space planning and used as a resource element for scientific control. Under the background of China’s territorial space planning, the protection and control of cultural landscape heritage in urban fringe areas and its overall environment are the focus of current research.

Urban fringe was first proposed in 1936 by German geographer, Herbert Louis, who found that originally rural areas had been gradually occupied by urban construction regions and had blended into urban areas during his study of Berlin urban structures. He then defined the zone as different from the type of urban land used as Stadtrandzonen.

In 1968, R. J. Pryor explained urban fringe as the fringe built-up areas and suburban areas with significant differences in urban-rural land use, society, and demography. Urban and rural areas are not well-delineated, bounded, or self-sufficient spaces [22]. During the period of rapid urbanization, the urban fringe area is the area where urban expansion occurs first, and land use change is the most active [23]. The urban fringe is quite unstable, influenced by urbanization activities, which leads to the strong dynamic fluctuations of multiple aspects such as economics, social structures, nature, and human landscape. With the expanding of urban areas and rapid development of the economy, urban fringes will be the first to be eaten away and deteriorated by urban areas, which therefore must be given importance in urban sustainable development. Major urban challenges facing China and Europe are related to changes in climate, environment, and to decision-making that makes urban and rural landscapes more susceptible to environmental pressures. Urbanisation is one of the most challenging processes we face in the 21st century. This calls for new thinking to create robust collaboration across urban–rural areasthat fosters sustainable development in both [24]. In Italy, a decisive steering role is entrusted to regional landscape planning, as introduced through the current Code of Cultural Heritage and Landscape. Regional landscape plans define the rules to which all municipal plans must conform [25].

K. E. Boulding et al. believe that more and more people have realized the importance of protecting cultural landscapes and thenatural environment under the increasingly serious situations of population explosion, rapid industry expansion, intensified natural resource consumption, ecological destruction, and the environmental pollution caused by the great industry and modern agriculture [26].

As cultural landscape heritage becomes part of the world’s heritage, its effective management has been causing concerns worldwide.In 1969, L. McHarg published Design with Nature, in which he discusses environmental issues from the perspectives of nature, history and humanity, and explains how natural procedure guides land development [6]. After decades of work, various countries have combined the regional protection of cultural heritage with the national and local development of culture and ecology, society, and other aspects, and they have also rationally set up relevant cultural heritage reserves. These measures, in advance, offer the basis for scientific decision-making and new ideas in the protection and management of closely relevant departments, such as urban planning, government management, and land use. Additionally, such measures have provided strong support for the strategy of regional overall coordinated development, and have gained much successful experience.

Sustainable urban planning is essential in mediating the natural and built environments globally, yet there is little progress as regards its attainment in developing countries [4]. With different national conditions, each country has its own strategy of the protection and use of cultural landscape heritage. For example, the U.S. was the first to establish the National Park System so as to maintain the non-profit, complete, scientific, and sustainable protection of its cultural landscape heritage. Unlike most Western developed countries, China has an urban-rural dualism where there are enormous differences between urban and rural areas. Besides, the fact that many cities remain in the process of urbanization has made urban fringe even more unstable. China’s large cultural landscape heritages are mostly located in urban fringe areas, which makes it easier for them to be influenced by the process of urbanization, and such special locations have given them special qualities different from other types of cultural landscape heritage. As a result, cultural landscape heritagesites in urban fringe areas are often developed into archaeological site parks with conservative features and historical memories, which can offer public cultural service and help the protection of relics as well as the strategy of rural vitalization, becoming a destination of cultural tourism and key supporter of beautiful rural construction.

However, since China has a behindhand archaeological site park construction, a mature model has not been formed in practice, and there are situations of “mass input with tiny output” in some cities’ archaeological site park construction. Meanwhile, the planning and construction of an archaeological site park requires the comprehensive consideration of land use, population distribution, industrial distribution, and matching public facilities with base facilities so as to promoting the rural-urban development by protecting historical sites. Hence, it is necessary to conduct research on the sustainable development efficiency of cultural landscape heritages.

In 2000, the UNESCO (United Nations Educational, Scientific, and Cultural Organization) proposed the Hoi An Protocols for Best Conservation Practice in Asia at the Bangkok conference, which stressed the inner links between tangible heritage, intangible heritage, and cultural landscape protection, and can serve as professional guidance to guarantee and maintain Asian cultural heritage sites in the context of Asian cultures. The European Landscape Convention has set out a much more holistic understanding of landscape than was previously the case within Europe. The Convention embodies thinking that is beginning to be reflected in the work of governments, environmental agencies and a wide range of interested parties within the landscape field in Europe. The European Landscape Convention helpfully does not value any one landscape above another, indeed it recognises that local and degraded landscapes are as likely to be of importance to the communities or cultures who visit them as those which are commonly found to be labelled as globally important.

In 2005, the ICOMOS (International Council on Monuments and Sites) held an international conference and passed the Xi’an Declaration, where they emphasized the importance of the environment to cultural heritage preservation in ever-changing cities and landscapes, and that the implementation of specific protection needs to consider the overall environment’s dual attributes of tangibility and intangibility [2].

The Chinese government attaches great importance to heritage preservation and has therefore published several national policy documents, including the Notice on Strengthening and Improving the Work of Cultural Relics, the Cultural Relics Protection Law of the People’s Republic of China, and The 14th Five-Year Plan for the Protection and Utilization of Great Sites. Besides, the Chinese government has also laid much focus on its intangible cultural heritage preservation, and published Interim Measures for the Protection and Management of National Intangible Cultural Heritage in 2006. This proves again that heritage is both tangible and intangible in Chinese traditional culture.

From the above, we suggest that sustainable development efficiency is suitable for assessing the current situation of cultural landscapes in urban fringes and predicting their potential for sustainable development. Sustainable development efficiency, referring to the ratio of resource elements’ effective gross output to the input, is also seen as the comprehensive reflection of effective allocation of factor input, running status, and operating management. The proper coordination of social economic development and ecological environment development is an inevitable requirement of sustainable development [27].

Since the concept of efficiency was proposed in 1957 by a British economist, who also put forward its measurement standard and model, efficiency study have stepped into a brand new stage. DEA (Data Envelopment Analysis), created by A. Charnes, W. W. Cooper and E. Rhodes in 1978, is the most widely used method in efficiency study [28,29,30,31,32,33,34,35]. DEA is a new field of crossover study involving operations research, management, and mathematical economics, which is a quantitative analysis method for evaluating the relative effectiveness of comparable units of the same kind using linear programming according to multiple input and output indicators.

At present, there have been many studies about applying DEA to efficiency studies, which therefore formed relatively normative research methods and ideas. For instance, Yang Li et al. studied the total factor energy efficiency of sustainable development in China based on DEA [36]. Zhu, S., Zhou, Z., Li, R. and Li, W. used the city-level panel data of the three urban agglomerations from 2006 to 2019 to construct the slacks-based measure integrating data envelopment (SBM-DEA) model for calculating each city’s carbon dioxide emission efficiency [37].

This study uses sustainable development efficiency as a tool of analysis, takes two cultural landscape sites in the Wuhan urban fringe as the objects of study, creates and sets a multi-dimensional evaluation based on the GIS-DEA-MI model, and conducts a comparative analysis on the differences and dynamic changes of the developmental efficiency of the above two cultural heritage sites. This method makes up for the deficiency of traditional subjective qualitative analysisand fits in the integrality of cultural heritage sites, so as to study the development efficiency of cultural landscape sites from a more objective and comprehensive perspective, to improve the overall sustainable development of tangible and intangible cultural heritage, and to provide a basis for early decision-making and post-implementation evaluation for the preservation and utilization of cultural landscape heritage under the background of urban renewal.

## 2. Materials and Methods

Many previous studies about development efficiency tended to use the DEA model only to study its non-spatial development efficiency, or to analyze spatial development efficiency solely by spatial aspects, such as land use efficiency, planning operational efficiency and urban updating efficiency, while urban issuesare compound, which requires comprehensive perspectives to summarize the overall development efficiency objectively and roundly. Additionally, such a study would better be designed as a comparative one, which can lead to precise knowledge of the differences of the overall resource utilization among different archaeological parks through comparing the development efficiency of the research objects so as to redefine their future directions and positions, adapt to new situations, and make reasonable decisions according to our new duties.

**Figure 1:**
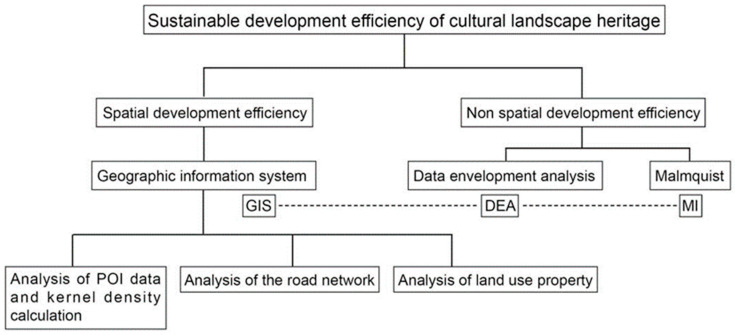
Knowledge mapping of the study on the developmental efficiency of cultural landscape heritage.

The evaluation of spatial efficiency is proceeded by assessing urban operational efficiency according to the planning and variation of urban space in other words, various types of urban construction land, while non-spatial efficiency is evaluated by attributive standards including urban natural environment, economy and society [38]. Therefore, the developmental efficiency of archaeological site parks consists of both spatial and non-spatial development efficiency, as shown in Figure 1.

A GIS (geographic information system), has strong capacities of analyzing and modeling geographic space as well as supporting making spatial decisions. POI (point of interest) data was imported to ArcGIS to generate core density diagrams, which can be used to calculate the density of the key factors in their surroundings so as to reflect the degree of concentration in certain ranges of POI distribution.

The DEA-MI model was chosen to study the non-spatial development efficiency of the above archaeological parks. The DEA (data envelopment analysis) model, created by A. Charnes, W. W. Cooper and E. Rhodes in 1978, has been widely used for evaluating projects and policies.

DEA, a non-parametric examination based on the concept of relative efficiency evaluation, in which the evaluated units and organizations are called the DMU (decision-making units), constructs a data envelope curve through selecting various input and output data of DMU, using linear programming, and taking optimal input and output as production frontiers.

In this method, the efficient point is on the leading surface and the efficiency value is set as 1, while the invalid point is on the outside, and is given a relative efficiency value standard within the range from 0 to 1.

The traditional DEA model can only offer longitudinal comparison of the production efficiency of decision making units at the same timing, while the DEA-Malmquist exponential model can investigate the dynamic variation of the efficiency of DMU in different stages, thus being able to analyze panel data with a relatively wide application.

To sum up, the evaluation based on the GIS-DEA-MI model can both be applied to the development efficiency of archaeological parks and other fields, such as evaluating urban and rural planning operational efficiency and evaluating urban updating efficiency. This method combines multiple research methods of management, geographic information systems, urban and rural planning, and other subjects to provide strategies and advice to improve the development efficiency of archaeological parks, optimize the planning of urban and rural space, and achieve the integrated development of urban and rural areas, and it also offers reference to the development efficiency of other archaeological parks.

## 3. Empirical Research

### 3.1. Research on Spatial Efficiency

#### 3.1.1. Analysis of POI Data and Kernel Density Calculation

POI (point of interest) data, based on the key data of location services, plays an essential role in urban planning studies and the preliminary planning of commercial development projects.

The distribution density of POI data is the expression of spatial phenomena, since human economic activities often manifest as the gathering of several joints and form gathering centers of different grades in different spatial statistics units. As a result, its kernel density can reflect the efficiency and potential of space development to some extent.

At present, research on the spatial efficiency of archaeological site parks still lacks a widely accepted standard. Professor Jonh Radke from the University of California, Berkeley, first put forward the theory of a two-step floating catchment, which was then improved by two scholars, Luo and Wang, who combined the gravity model method and the floating catchment area method. The former calculates the service potential of housing estates, while the latter calculates the availability of public service facilities, and the combination is named 2SFCA (two-step floating catchment area method). This method can be used to study the distribution features of the population and public service facilities in different searching areas through constructing different motion states of people in cities and the reachable range finitely formed by the service capacity of urban public service facilities as the searching areas, and to further measure the opportunities people seize to enjoy public service [39]. It can be expressed by mathematical expressions such as Equation (1).

(1) $$A_{i}^{F}=\underset{j=\left\{d_{ij}\leq d_{0}\right\}}{\sum }R_{j}=\underset{J\in \left\{d_{ij}\leq d_{0}\right\}}{\sum }\left(\frac{S_{j}}{\sum_{k\in \left\{d_{ij}\leq d_{0}\right\}}D_{k}}\right)$$

One of the weaknesses of this method is that it uses the same searching radius for all the facilities and citizens. Therefore, McGrail put forward the idea of a dynamic radius according to differences in population density in different areas in 2014, and it was discovered through many experiments that increasing the searching radius gradually is feasible with the decrease of population density. The Huangpi District, where Panlongcheng is situated, and the Jiangxia District, where the Tomb of the King of the Ming Dynasty is situated, have significantly less population density than central areas, so there should be a larger searching radius. By praxiology, the walking speed of an adult is about 5 km/h, whose normal maximum psychological endurance is 30 min, calculated as 2.5 km.

Meanwhile, with an archaeological site park as our research object, which has a larger coverage and more service objects, including local and non-local visitors apart from its community residents, we also need to consider the searching radius under non-walking conditions.

According to big data, Liusong conducted research on the service radius of community parks in Shanghai based on the signaling data of cell phones, which indicated that the average was 5879 m long [40]. Additionally, an archaeological site park has a significantly larger scale than normal parks, so a small range of a study may fail to objectively reflect the surrounding infrastructure and space characteristics. In conclusion, the range of this research was considered appropriate within a 5 km radius centered in the park.

By adding the POI data of Wuhan in 2020 to ArcGIS, and conducting research within the range of 5 km centered, respectively, on Panlongcheng and the Tomb of the King of the Ming Dynasty, the POI data of the above two objects, including the surrounding traffic facilities, public facilities and service facilities, were calculated to investigate kernel density and were analyzed according to their current situation.

**Figure 2:**
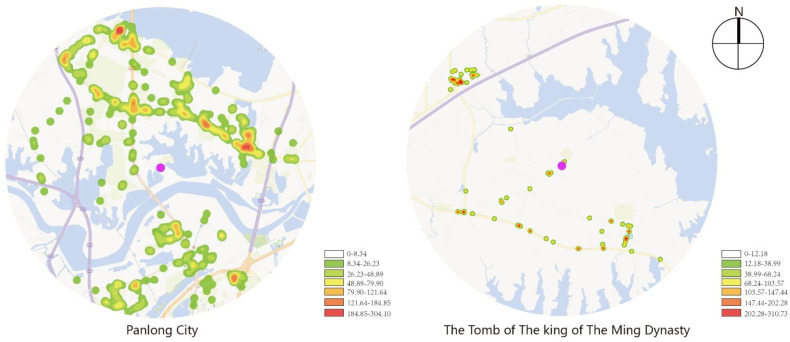
Kernel density analysis of traffic facilities.

As shown in Figure 2, the traffic facilities surrounding Panlongcheng are uniformly distributed and are densely distributed in various spots, while that of the Tomb of the King of the Ming Dynasty are mainly concentrated on the north side of the highway, connecting Shanghai and Chongqing, which gathers cross-border e-commerce industries, and along Fenglian Avenue, and no dense distribution has formed.

**Figure 3:**
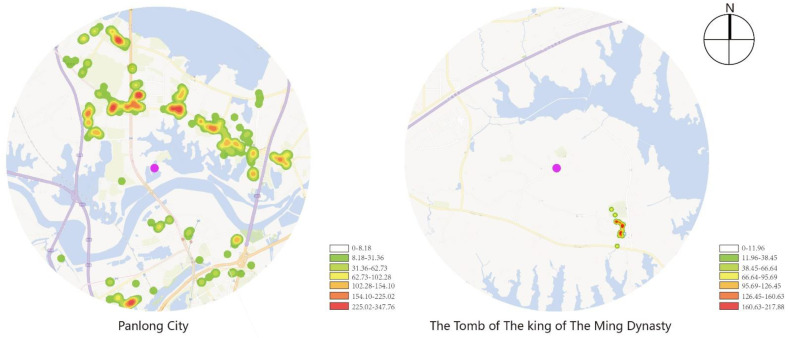
Kernel density analysis of communal facilities.

As shown in Figure 3, the traffic facilities surrounding Panlongcheng are relatively uniformly distributed, but no large-scale distribution has formed in most districts except in Outlets Plaza near Houhu Bridge. With few public facilities nearby, the public services of the Tomb of the King of the Ming Dynasty are mainly its archaeological station and village committee, which serve a quite limited range of areas where the objects are mostly local citizens.

**Figure 4:**
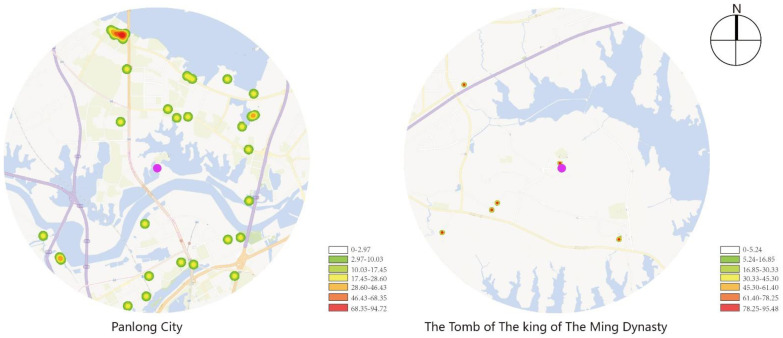
Kernel density analysis of service facilities.

Figure 4 indicates that service facilities near Panlongcheng are mainly north-south distributed with relatively few service facilities in the west part, but they are uniformly distributed on the whole, and have formed dense distributions in multiple areas, which reveals relatively well-equipped service facilities. As for the Tomb of the King of the Ming Dynasty, its surrounding service facilities are near Wuhan Qingchuan University in the southeast part, which serve mostly teachers and students in the school with a limited range of service.

#### 3.1.2. Analysis of the Road Network

Traffic is the precondition of the development of an archaeological site park, and its convenience reflects the potential of space development. The study modified the road network analysis according to A Map of Wuhan Planning, respectively, centered in the two research objects within the range of the 5 km radius.

**Figure 5:**
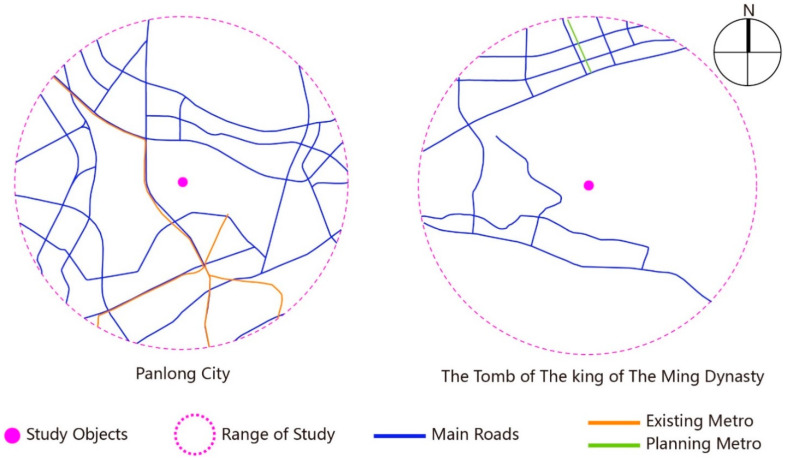
Analysis of road network.

Figure 5 proves that road facilities near Panlongcheng have been relatively well-equipped and are distributed uniformly. There are three rail traffic lines in the range of research, and 37 intersections in main roads. When it comes to the Tomb of the King of the Ming Dynasty, whose road facilities have notbeen fully constructed and uniformly distributed, there is no rail traffic, and its main roads have 19 intersections. According to such an analysis of the road network, obviously, Panlongcheng has a better-built road network than the Tomb of the King of the Ming Dynasty with higher space accessibility. In 2019, the construction of Line 19 of the rail traffic in Wuhan was officially started, which connects Wuhan Railway Station and the Optical Valley Bonded Zone, significantly increasing the accessibility of the Tomb of the King of the Ming Dynasty Archaeological Park.

#### 3.1.3. Analysis of Land Use Property

A Map of Wuhan Planning, published by Wuhan Natural Resources and Planning Bureau, specified the nature of the land use in Wuhan, and it is a reflection of how the space in a district develops and its potential. It is based on this map that the the figure of the analysis of land use property is modified.

**Figure 6:**
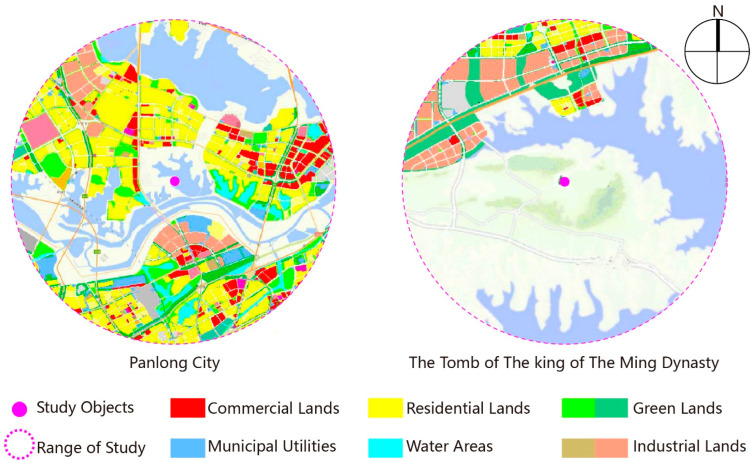
Analysis of land use property.

As seen from Figure 6, Panglongcheng has abundant land use properties, which are mainly used for residents and businesses, while the Tomb of the King of the Ming Dynasty has planning for land use property in the north part only, which is mainly for industrial use.

The efficiency of space development and utilization near Panlongcheng is significantly higher than that of the Tomb of the King of the Ming Dynasty. With Longquan Mountain, Liangzi Lake and other natural resources nearby, the development and utilization of the Tomb of the King of the Ming Dynasty should fulfil the principle of ecology first, lay a focus on ecological protection, and develop some businesses, industries and logistics only in the northern comprehensive bonded areas. Its unique strength in ecology helps to carve out a different and distinctive path of development than that of Panlongcheng.

The land use property near the Tomb of the King of the Ming Dynasty can be adjusted according to the space planning of national land, and it will benefit ecological protection and the longterm development of archaeological parks.

The efficiency of space development largely depends on the spatial pattern of urban and rural areas, such as the density of the road network, the properties of construction land in urban and rural areas, and the distribution of public service facilities. The application of GIS can directly reflect these features, which benefit the reasonable arrangement and adjustment of construction land in urban and rural areas, further improvement of equipment, and the cultivation of physical space for urban industries, policy measures and other immaterial planning objects.

### 3.2. Research on Non-Spatial Efficiency

#### 3.2.1. The Selection of the Index

The evaluation index of the development efficiency of archaeological site parks still lacks a common standard, but it can be roughly classified into the input index and the output index. The selection of the index should be scientific, which means the input index should be logically related to the output index ona quantitative basis [41,42,43,44].

As is shown in Table 1, five indexes were selected in this study, including the general income and general reception of tourism as the output indexes, and industrial structures (ratio of tertiary industries), public utility input and the investment in human capital as the input indexes. Besides, the DEA method demands that DMU should be twice as large as the sum of output indexes and input indexes, thus the study is compliant since the DMU counted as 12. Table 1 presents how the indexes were selected and quantified.

The data comes from the Statistical Yearbook of Hubei Province, the Statistical Yearbook of Wuhan, and online open-source data over the ten years from 2010 to 2019. Affected by the COVID-19 pandemic, the data of tourism since 2020 has dramatic fluctuations and was therefore not involved in this research.

#### 3.2.2. Data Analysis

**Figure 7:**
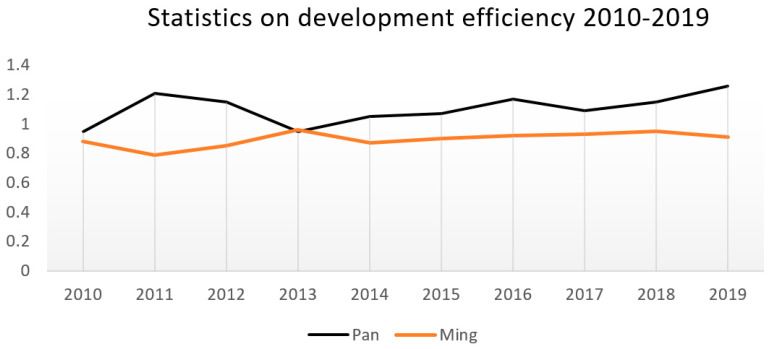
Statistical graph of non-spatial development efficiency.

Statistical data from 2010 to 2019 was added to Deap2.1 and the results are presented in Table 2, which can be drawn into a line chart as in Figure 7.

Generally, Panlongcheng and the Tomb of the King of the Ming Dynasty share a relatively high development efficiency, with an average value above 0.8, and they have small standard deviations of tourism efficiency over the years. The standard deviation of Panlongcheng Archaeological Park is about 0.09, while that of the Tomb of the King of the Ming Dynasty is about 0.04, but both of their development efficiencies narrows the distinction, and the standard deviation decreases year by year.

The results accord with the socio-economic development of Wuhan, where the archaeological park of the Tomb of the King of the Ming Dynasty in the Jiangxia District has relatively deficient and late-constructed foundations, and its development efficiency over the years has been left behind by Panlongcheng Archaeological Park; on the other hand, however, because Panlongcheng Archaeological Park has been primarily constructed and its relevant equipment has been implemented, its development efficiency has small fluctuations in general. In recent years, with the smooth implementation of the archaeological work of the Tomb of the King of the Ming Dynasty and the increasing investment in various aspects, its development efficiency is increasing, narrowing the gap between it and Panlongcheng Archaeological Park.

**Figure 8:**
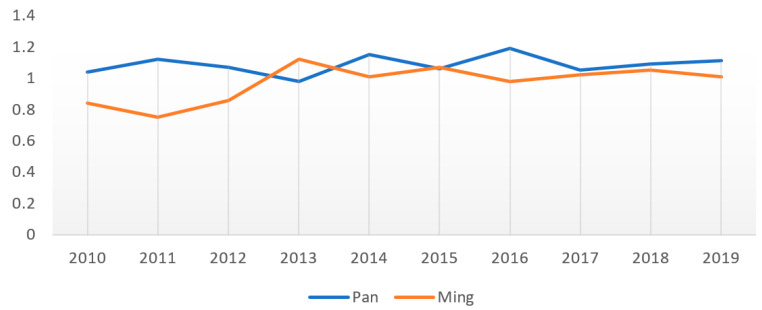
Statistical graph of the degree of change in non-spatial development efficiency.

Based on the results of the above tourism efficiency from 2010 to 2019, MI method is applied to calculate the variation of two parks’ non-spatial development efficiency to more objectively and precisely picture the dynamic variation of the non-spatial development efficiency. The results are shown in Table 3, and its data is drawn into another line chart, as shown in Figure 8.

The above data show that there has been a distinctive difference between the non-spatial development efficiency of Panlongcheng and the Tomb of the King of the Ming Dynasty. Over the past ten years, the MI of Panlongcheng Archaeological Park had an average standard deviation of 0.05, while that of the archaeological park of the Tomb of the King of the Ming Dynasty was 0.11, but both showeda main feature of increase. The non-spatial development efficiency of the two parks varied in accordance with their socio-economic development.

## 4. Conclusions

This study constructs a multidimensional evaluation based on GIS-DEA-MI, which was used to conduct research on the spatial and non-spatial sustainable development efficiency of the two cultural landscape heritagesites Panlongcheng Archaeological Park and the Tomb of the King of the Ming Dynasty, in the urban fringe of Wuhan, China. According to the study, we are able to conclude that:

1. Seen from spatial development efficiency, Panlongcheng has a much higher core density of POI data than the Tomb of the King of the Ming Dynasty, and the quantity and density of its road network are also superior than the latter’s. Hence, more effort should be put into the construction of the Tomb’s surrounding basic facilities. As for the type of land use, Panlongcheng has more varied types of its surrounding land use, which has been involved in Wuhan’s urban-rural land use planning, while the surrounding land types of the Tomb are mostly ecological agriculture and forestry, with the obvious advantage of natural resources. Consequently, future work of urban planning and cultural landscape heritage preservation and utilization should stress the improvement of the Tomb’s surrounding land use planning and the protection of its environmental integrality, so as to facilitate its sustainable and green development with its advantage in natural resources.
2. The non-spatial development efficiency of the two parks from 2010 to 2019 had small fluctuations, but that of the archaeological park of the Tomb of the King of the Ming Dynasty was low in general. As a national archaeological park with much better policy support, investment and human resources, Panlongcheng Archaeological Park enjoyed higher non-spatial development efficiency than the Tomb. As a result, the improvement of the Tomb’s non-spatial development efficiency should be achieved by digging deep into its cultural value, actively developing its protection and declaration of the world’s cultural heritage, and seeking stronger support in policy, fund, and manpower.
3. The application of the GIS-DEA-MI model in research on the development efficiency of archaeological site parks in urban fringes is appropriate both in the early planning of construction and in the post-assessment stage. The study of the early planning can decide reasonable input and optimize the investment efficiency, while the post-assessment stage helps to improve resource allocation. Local government can make macroscopic adjustments according to the dynamic variation data of tourism efficiency to promote the development efficiency of archaeological parks in urban fringe and the harmonious development of urban and rural areas.

What remains to be further studied and discussed may include:

4. Research on spatial development efficiency still has room for a systematic study combining the theory of national land planning;
5. Research on non-spatial development still lacks common and widely accepted standards for the selection of output indexes and input indexes.

## Figures and Tables

**Table 1:** Evaluation index and quantitative standard.

Evaluation Index	Quantitative Criteria	Units
Total income from tourism	Annual tourism revenue of the administrative region	CNY 100 million
Total tourist arrivals	Annual tourism reception of the administrative region	Person-time
Industrial structure	Proportion of tertiary industry	percentage
Public service input	Annual total input of public utilities in the administrative region	CNY 100 million
Human capital input	Total number of tertiary industry employees	Person

**Table 2:** Results of non-spatial development efficiency.

	2010	2011	2012	2013	2014	2015	2016	2017	2018	2019	Mean Value
Pan	0.95	1.21	1.15	0.95	1.05	1.07	1.17	1.09	1.15	1.26	1.21
Ming	0.88	0.79	0.85	0.96	0.87	0.90	0.92	0.93	0.95	0.91	0.89

**Table 3:** Degree of change in non-spatial development efficiency.

	2010	2011	2012	2013	2014	2015	2016	2017	2018	2019	Mean Value
Pan	1.04	1.12	1.07	0.98	1.15	1.06	1.19	1.05	1.09	1.11	1.09
Ming	0.84	0.75	0.86	1.12	1.01	1.07	0.98	1.02	1.05	1.01	0.97

## Data Availability

The data presented in this study are available on request from the corresponding author.
